# Poly(ε-Caprolactone)/Poly(Lactic Acid) Blends Compatibilized by Peroxide Initiators: Comparison of Two Strategies

**DOI:** 10.3390/polym12010228

**Published:** 2020-01-16

**Authors:** Marta Przybysz-Romatowska, Józef Haponiuk, Krzysztof Formela

**Affiliations:** Department of Polymer Technology, Faculty of Chemistry, Gdansk University of Technology, Gabriela Narutowicza 11/12, 80-233 Gdansk, Poland; jozef.haponiuk@pg.edu.pl

**Keywords:** poly(ε-caprolactone), poly(lactic acid), reactive blending, peroxide initiators, copolymers, compatibilization

## Abstract

Poly(ε-caprolactone) (PCL) and poly(lactic acid) (PLA) blends were compatibilized by reactive blending and by copolymers formed during reaction in the solution. The reactive blending of PCL/PLA was performed using di-(2-tert-butyl-peroxyisopropyl)benzene (BIB) or dicumyl peroxide (DCP) as radical initiator. PCL-*g*-PLA copolymers were prepared using 1.0 wt. % of DCP or BIB via reaction in solution, which was investigated through a Fourier transform infrared spectrometry (FTIR) and nuclear magnetic resonance (NMR) in order to better understand the occurring mechanisms. The effect of different additions such as PCL-*g*-PLA copolymers, DCP, or BIB on the properties of PCL/PLA blends was studied. The unmodified PCL/PLA blends showed a sea-island morphology typical of incompatible blends, where PLA droplets were dispersed in the PCL matrix. Application of organic peroxides improved miscibility between PCL and PLA phases. A similar effect was observed for PCL/PLA blend compatibilized by PCL-*g*-PLA copolymer, where BIB was used as initiator. However, in case of application of the peroxides, the PCL/PLA blends were cross-linked, and it has been confirmed by the gel fraction and melt flow index measurements. The thermal and mechanical properties of the blends were also investigated by means of differential scanning calorimetry (DSC), thermogravimetric analysis (TGA), dynamic mechanical analysis (DMA), and tensile strength.

## 1. Introduction

The preparation of innovative materials is associated with their continuous modification and improvement, thanks to which they may meet high application requirements. The most common methods of modification are blending and cross-linking of polymers [1,2,3]. Biodegradable polymer blends give a wide range of possibilities to obtain materials with properties that would not be possible using homopolymers without losing their biodegradability [4]. However, the final properties of polymer blends, achieved by physical mixing, may be disadvantageous or not observable at all. This is due to their thermodynamic incompatibility associated with low free energy mixing [5,6,7]. In order to successfully mix two incompatible polymers, thus improving their functional properties and broadening their use, it is necessary to introduce additional stimuli/factors, e.g., organic peroxides [8]. With their inclusion under the influence of high temperature, we obtain free radicals, thanks to which it is possible to (a) create copolymers of polymers used by combining two different macro-radicals, (b) cross-link of individual components, and (c) degrade of material more susceptible to peroxides. It is worth mentioning that, in the currently published research papers, among the low-molecular-weight additives, peroxides are one of the commonly used agents to improve properties of biodegradable polymer blends, having dicumyl peroxide [9,10] and 2,5-dimethyl-2,5-di-(tertbutylperoxy)-hexane organic peroxide [11,12] as free-radical initiators, while application of other organic peroxides is rather limited and concerns mainly single biodegradable polymers, not their blends [13,14,15]. It is well known that initiator type and concentration of free radicals, as well as reactive blending conditions, strongly affect the formation of new bonds between polymer chains. Semba et al. [16] studied the influence of dicumyl peroxides (DCP) containing 0, 0.1, 0.2, and 0.3 phr on PLA/PCL blends’ properties. The blends were prepared with various weight ratios of 100/0, 70/30, and 0/100 and were compounded in a twin-screw extruder. Garcia-Garcia et al. [17] reported the influence of dicumyl peroxide (DCP) in the range 0–1 wt. % on the properties of poly(3-hydroxybutyrate) (P3HB) and poly(ε-caprolactone) (PCL) blends at a fixed weight ratio equal to 75/25. The results showed that the addition of 1 wt. % of DCP to PHB/PCL blends was adequate and had a positive effect on their compatibility and mechanical properties. Ma et al. [18] tried to improve the toughness of PLA by blending it with 20 wt. % PBAT. This blend showed the elongation at break of around 200% with no significant improvement in impact strength (60 J/m). This drawback was successfully corrected by compatibilization with optimal amounts of DCP concentration (e.g., 0.5 wt. %).

As stated before, the two polymers can form block copolymers in addition to crosslinking/branching reactions. The branched/crosslinked structure was demonstrated by the solid-like behavior of the material at lower frequency rheological properties. The formed PLA-*b*-PBAT copolymer was acting as compatibilizing agent to improve the adhesion between the PBAT and PLA. Coltelli et al. [11] selected liquid peroxide, e.g., 2,5-dimethyl-2,5-di(tert-butylperoxy)hexane, to compatibilize the PLA/PBAT blend. The PLA/PBAT (75/25 wt. %) blend with 0.2 wt. % of peroxide showed fine morphology with reduced inclusion phase size. As a result, the elongation at break of the compatibilized PLA/PBAT was higher than the corresponding uncompatibilized blend. Poor processability, low melt-strength, and insufficient impact toughness of the fully biobased and biodegradable PHB/PLA blend are corrected after reactive compatibilization with DCP [19]. The compatibility of this reactive blend improved the formation of branching/cross-linking structures at the interface and improved compatibility between the phases resulted in reduced inclusion PLA. 

In the present paper, the biodegradable PCL/PLA blends in weight ratio of 75/25 were compatibilized by two strategies: (i) reactive blending using DCP or BIB as free-radical initiators and (ii) using synthetized PCL-*g*-PLA copolymers via melt blending process. The effects of the reactive processing of PCL/PLA blends using organic peroxides and compatibilization of the blends by adding PCL-*g*-PLA copolymers aiming to improve morphology and properties were measured. The results of conducted studies allowed to compare those two methods of modification of PCL/PLA blends.

## 2. Materials and Methods 

### 2.1. Materials

Poly(ε-caprolactone) (PCL) (Capa™ 6800) of density 1.14 g/cm^3^ and melt flow index of 7.3 g/10 min (190 °C/2.16 kg) was obtained from Perstorp Holding AB (Malmö, Sweden), and poly(lactic acid) (PLA) (Ingeo™ 3251D) of melt flow index of 35 g/10 min (190 °C/2.16 kg), and density of 1.24 g/cm^3^ was purchased from NatureWorks LLC (Minnetonka, MN, USA). Dicumyl peroxide (DCP) (*T*_1/2_ = 0.1 h at 154 °C) and di-(2-tert-butyl-peroxyisopropyl)-benzene (BIB) (*T*_1/2_ = 0.1 h at 156 °C) were obtained from Pergan Peroxides Company (Quedlinburg, Germany).

### 2.2. Sample Preparation

#### 2.2.1. Preparation of PCL-*g*-PLA Copolymers during Reaction in Solution

The composition of PCL/PLA (3/1) was placed in a three-necked round bottom flask equipped with a reflux condenser and thermometer and dissolved in xylene at 140 °C. The solution was stirred using a magnetic stirrer. Then, the initiator DCP or BIB (1.0 wt. %) was added, and the mixture was stirred for 1 h. The solution was precipitated in a beaker with cold methanol and subsequently filtered. The samples ware dried for 48 h at room temperature. Then the copolymers were used as compatibilizers for PCL/PLA blends in melt state blending. Samples compatibilized with PCL-*g*-PLA copolymers were coded as: PCL/PLA/DCP_comp._ and PCL/PLA/BIB_comp._ for DCP and BIB initiators, respectively. 

#### 2.2.2. Preparation of PCL/PLA Blends via Reactive Blending 

The blends of PCL/PHB were prepared using the Brabender internal mixer (Brabender^®^ type GMF 106/2) at a temperature of 170 °C, where a rotor speed was set to 100 rpm for a total time of 8 min. The samples containing an amount of DCP or BIB were 1.0 wt. % or 5 wt. % of copolymers. The weight ratio was set at 75/25 wt. %. The PCL/PLA blends were premixed into mixing chamber for 4 min; then, the reactive reaction using DCP or BIB followed, and compounding continued for the next 4 min. Samples of all the compositions have been molded to 2 mm thickness by the pressing method under pressure of 4.9 MPa at 170 °C for 1 min and then at room temperature for 5 min. Samples compatibilized by reactive blending were coded as: PCL/PLA/DCP and PCL/PLA/BIB for DCP and BIB initiators, respectively.

### 2.3. Characterization of PCL/PLA Blends

Fourier transform infrared spectroscopy (FTIR) analysis of neat PCL, PLA, and PCL-*g*-PLA were recorded with a Nicolet Spectrometer IR200 spectrometer from Thermo Scientific (Waltham, MA, USA). The device had an ATR attachment with a diamond crystal. Measurements were performed with 1 cm^−1^ resolution in the range from 4000 to 400 cm^−1^ and 64 scans. 

The PCL-*g*-PLA copolymers that were prepared in solution reaction, were dissolved in deuterate CDCl_3_, and ^1^H NMR spectra were recorded at 200 MHz nuclear magnetic resonance (NMR) spectrometer (Gemini 200, Varian, Palo Alto, CA, USA). Spectra were analyzed using MestreNova software (Mestrelab Research, S.L., Santiago de Compostela, Spain). 

The thermal behavior and crystallization of the samples were measured by differential scanning calorimetry (DSC); measurement was carried out on a DSC 204 F1 Phoenix apparatus (NETZSCH-Gerätebau GmbH, Selb, Germany). The PCL/PLA samples of 4–6 mg weight were placed in aluminum pan and heated from −80 °C to 200 °C under N_2_ atmosphere at a rate of 10 °C/min. The DSC thermograms with the melting points (*T*_m_), crystallization temperature (*T*_c_), enthalpy of cold crystallization and enthalpy of melting were recorded during the second heating. For all the samples, the degrees of crystallization of PCL phase (*X*_cPCL_) and PLA phase (*X*_cPLA_) were calculated according to the following Equation (1):

(1) $$degree\;of\;crystallinity\;(X_{c})=\left(\frac{\Delta H_{m}}{\Delta H_{0}\times W_{f}}\right)\times 100\%$$

where Δ*H*_m_ is the specific melting enthalpy, Δ*H*_0_ is the melting enthalpy of 100% crystalline virgin polymer (where the melting enthalpy of 100% PCL is 136 J/g [20] and 93.7 J/g for PLA [21]), and *W*_f_ is the weight fraction of PCL or PLA phase in PCL/PLA blends. 

Thermogravimetric analyses (TGA) were carried out using the model Q600 from TA Instruments (New Castle, DE, USA). A total of approximately 10 mg of PCL/PLA blends were heated from 25 to 750 °C with a temperature increase rate of 20 °C/min in an inert gas atmosphere—nitrogen with a flow rate of 100 mL/min. 

Dynamic mechanical analysis (DMA) of the PCL/PLA blends were investigated using a DMA Q800 from TA Instruments (New Castle, DE, USA). The samples in the form of strips (40 mm × 10 mm × 2 mm) were measured in single cantilever mode at a constant frequency of 10 Hz as a function of temperature from −100 °C to 80 °C a heating rate of under nitrogen flow. 

The mechanical properties of the PCL/PLA blends were measured with standard dimensions according to ASTM638 at room temperature using a Zwick Z020 tensile tester (Ulm, Germany) equipped with a 20 kN load cell and crosshead speed of 50 mm/min. 

Results of static and dynamic mechanical analysis were used to calculate the brittleness parameter of investigated materials, in accordance with the following formula proposed by Brostow et al. [22] Equation (2):

(2) $$B=\;\frac{1}{\varepsilon_{b}\times E^{\prime }}$$

where *B* is the brittleness parameter, 10^10^ %·Pa; *ε_b_* is the elongation at break, %; *E’* is the storage modulus at 25 °C, MPa.

The stress-strain values are taken from an average of five repeats. The hardness of PCL samples was determined via Shore’a method in accordance with ISO 868 at room temperature, using a Zwick 3131 hardness tester (Ulm, Germany). All the values were expressed in ShD and the measurement was repeated ten times for each sample. 

Gel fraction was measured by the weight remaining after dissolving the sample in the solvent chloroform using the following Equation (3):

(3) $$\;\;Gel\;fraction\;\left(\%\right)=(W_{g}/W_{0})\times 100$$

where *W*_0_ is the dry weight of the PCL/PLA sample, *W*_g_ is the dry gel component of the PCL/PLA sample after being dissolved in the solvents at room temperature for 72 h. 

Melt flow index (MFI) of PCL/PLA blends was determined at 170 °C, with load of 2.16 kg, according to ISO 1133, using Mflow plastometer from Zwick (Ulm, Germany).

## 3. Results

### 3.1. Grafting/Branching of PCL and PLA Polymers via Peroxide Initiators

**Scheme 1:**
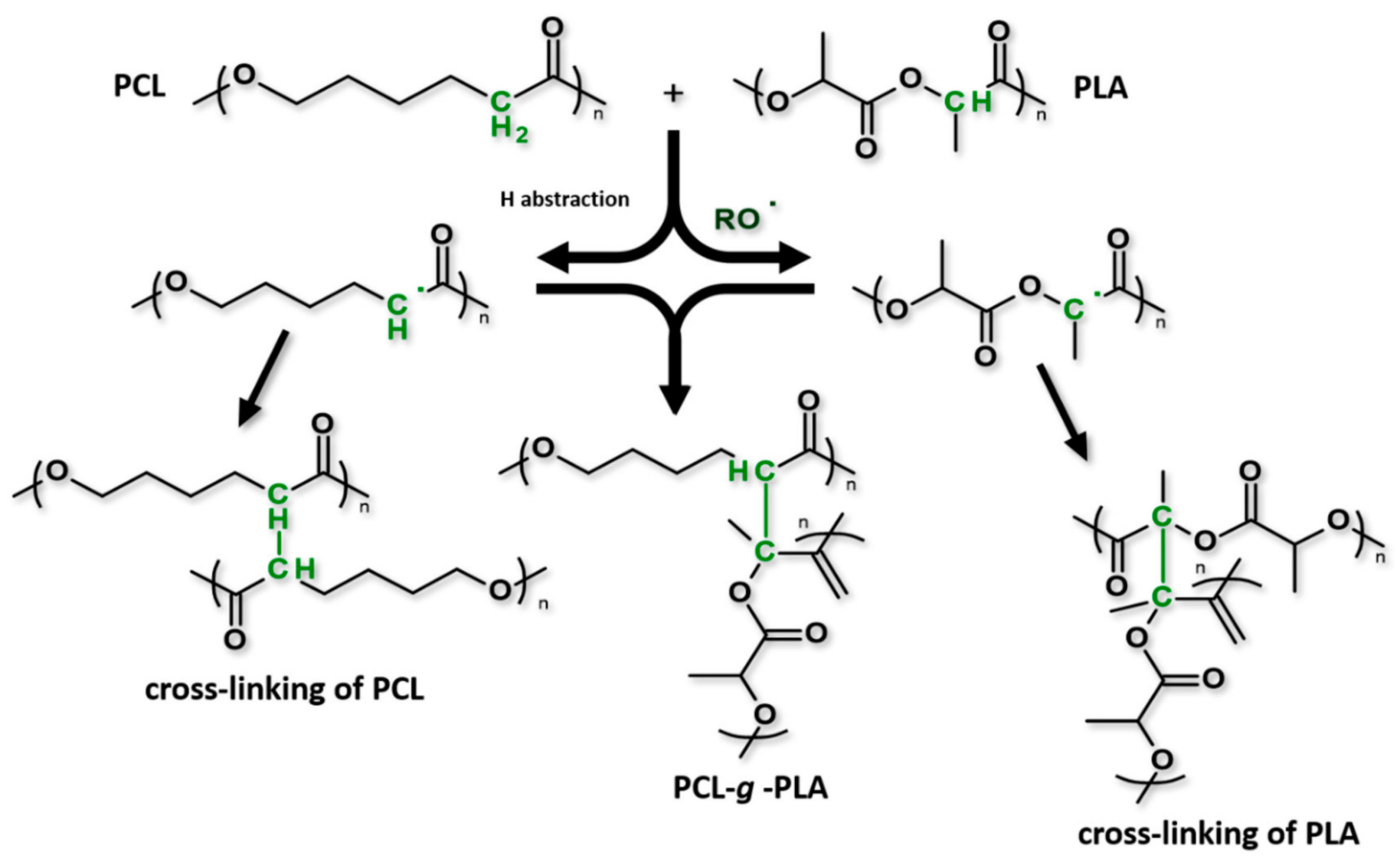
Likely reaction pathway for the cross-linking or grafting reaction of PCL and PLA in the presence of the free-radical initiator.

The modification of PCL and PLA was carried out in two ways: in-situ via reactive blending and in solution, in order to obtain pure materials and for a more accurate explanation of their structure and reaction mechanism. Scheme 1 presents the proposed reaction pathway for the cross-linking or grafting reaction of PCL and PLA in the presence of the free-radical initiator. Our assumption included preparation of PCL-*g*-PLA copolymers in solution, where DCP or BIB were used as initiators. Conducting the reaction at 140 °C for 1 h allowed a slower decomposition of peroxides, resulting in formation of the copolymers. As a step two, the materials obtained in solution were also be applied as compatibilizers to appropriate polymer systems, and efficiency of their compatibilization was determined.

During reactive processing at 170 °C, the decomposition of peroxides occurs more rapidly, and the cross-linking of individual polymers or their blends happens more easily, which can also improve morphologies of the blends. Moreover, the free radicals can also lead to chain *β*-scission and degradation of PCL or PLA or both. Although the degradation is not beneficial, the short polymer chains in the blends can act as plasticizers, what might reduce the tension in the PCL/PLA phases boundary.

### 3.2. Chemical Structure of PCL-g-PLA Copolymers Prepared in Solution

**Figure 1:**
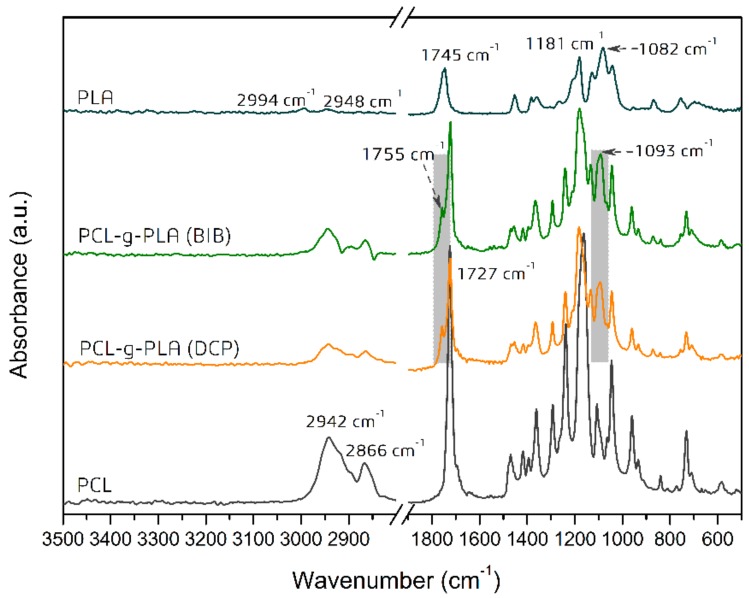
FTIR absorption spectra of neat PCL and PLA, and PCL-*g*-PLA copolymers.

The PCL-*g*-PLA copolymers were obtained using 1.0 wt. % DCP or BIB as a radical initiator in solution and coded as PCL-*g-*PLA (BIB) and PCL-*g*-PLA (DCP). The reaction was conducted in xylene at 140 °C for 1 h from the moment of DCP or BIB adding. The copolymers were prepared at a weight ratio of 3:1 of PCL:PLA. During the reaction in solution, organic peroxides generate free radicals, which may lead to production of macro-radicals of PCL and PLA and the creation of copolymers. Figure 1 shows the FTIR spectra of PCL-*g*-PLA copolymers compared with the neat PCL and PLA, to examine the creation of copolymers using DCP or BIB. The FTIR spectrum of neat PCL showed the characteristic absorption bands corresponding to C=O stretching vibration at 1727 cm^−1^, the CH_2_ stretching at 2942 cm^−1^ and 2866 cm^−1^. The spectrum of pure PLA showed absorption bands corresponding to C=O stretching vibration at 1745 cm^−1^, CH_3_ stretching at 2994 cm^−1^ and 2948 cm^−1^, and C–O–C stretching, the symmetric band in the range of 1079–1181 cm^−1^, the symmetric bending of the C–H group at 1360 cm^−1^, the symmetric bending of –CH_3_ at 1451 cm^−1^. The FTIR spectra of PCL-*g*-PLA copolymers showed the main absorption bands were shifted up a little to wavenumbers at 1755 cm^−1^ (v OC=O) and 1093 cm^−1^ (v C–OH). Moreover, the chain branching formation between PCL and PLA polymers is possibly overlapped by the C–O–C band corresponding to PLA (1079–1181 cm^−1^).

**Figure 2:**
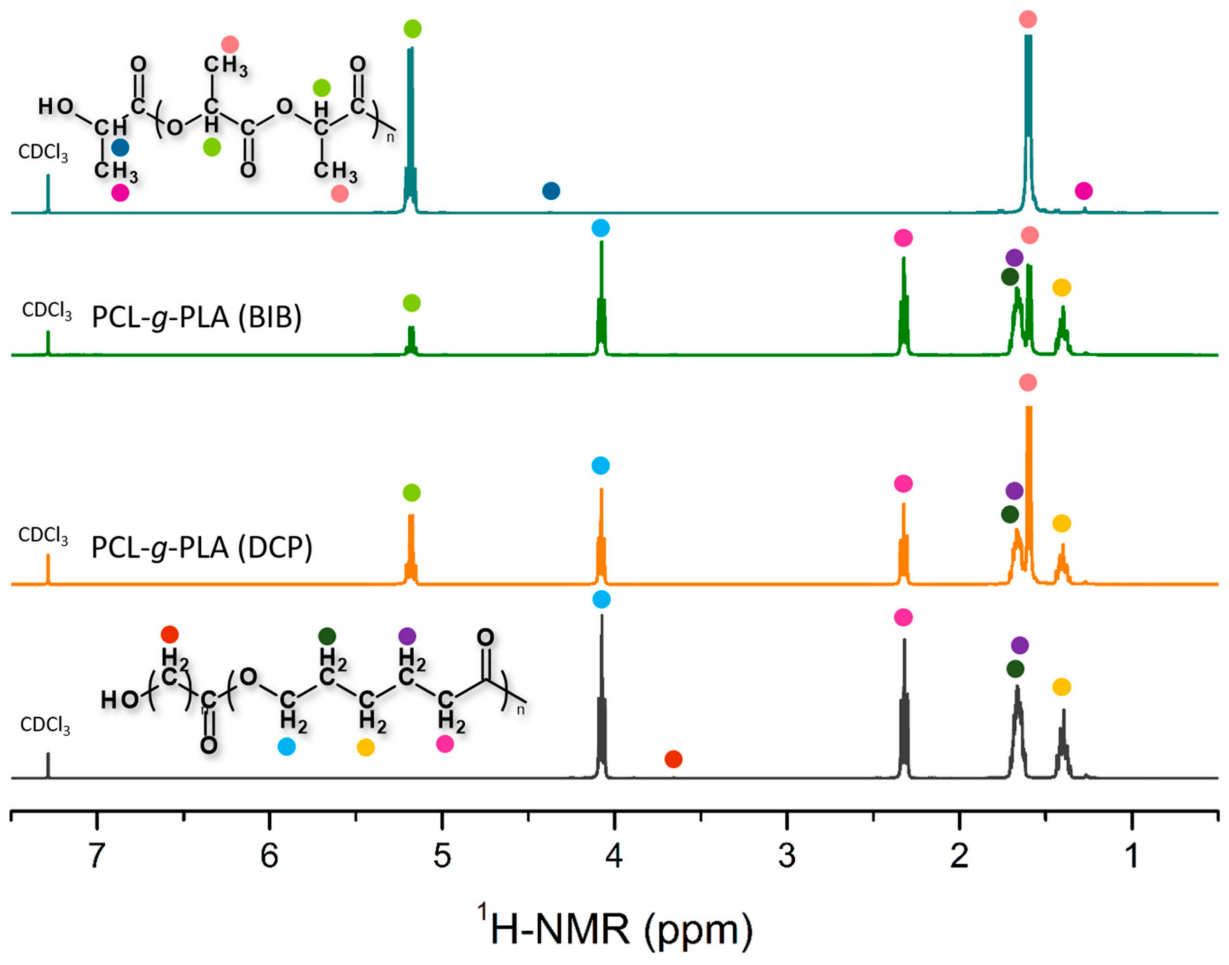
^1^H NMR spectra of neat PCL and PLA, and PCL-*g*-PLA copolymers.

The ^1^H NMR spectra of neat PCL and PLA, as well as PCL-*g*-PLA copolymers in deuterated chloroform (CDCl_3_) can be observed in Figure 2. In the ^1^H NMR spectra, the two typical peaks at ● 1.60 ppm and ● 5.18 ppm are associated with the hydrogens of CH_3_ and CH from PLA, respectively. The peak at 7.2 ppm is attributed to the deuterated chloroform used as solvent. In the ^1^H NMR spectra, signals are assigned as follows: ● 4.08 ppm, ● 2.32 ppm, ●● 1.39 ppm, and ● 1.66 ppm, which characterize the polymer chain of PCL. The peak position of methylene proton in the main chain of grafted PLA could be overlapped with the methylene proton peak of *β*- and *δ*-carbon atoms of PCL; therefore, new signals confirming grafting cannot be observed. 

### 3.3. Morphology of PCL/PLA Blends

**Figure 3:**
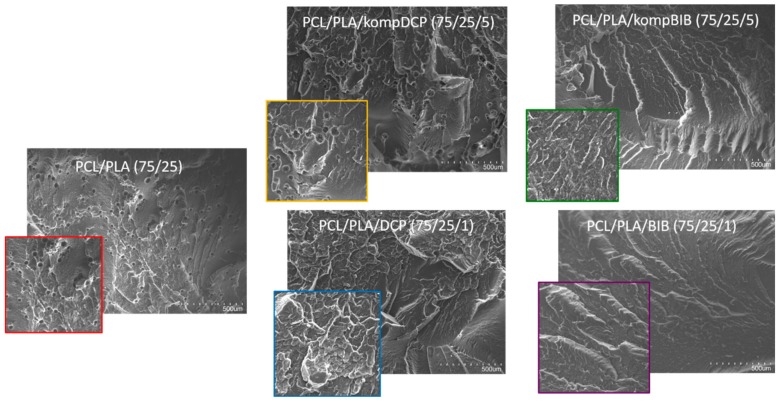
SEM images of PCL/PLA blend (75/25), and PCL/PLA blends compatibilized by copolymers and DCP or BIB in reactive blending.

The morphology of PCL/PLA blend, unmodified and compatibilized by PCL-*g*-PLA or reactive blending, was observed by the scanning electron microscopy (SEM). Figure 3 shows the SEM images of cryo-fractured surface of the physical blend, PCL/PLA (75/25) compatibilized by PCL-*g*-PLA copolymers, and modification by organic peroxides. The obvious difference between the PCL and PLA phase in the physical PCL/PLA (75/25) blend can be observed. It indicated that PCL and PLA in nonmodified blends were not compatible. For the PCL-rich blend without organic peroxide phase, separation and irregular PLA dispersion are observed. In this case, the size of the domains of PLA changes after the modification. The images of SEM display the PCL matrix and PLA dispersed domains, which are mostly spherical, this corresponds to a typical sea-island morphology. The PCL/PLA blends modified by DCP or BIB peroxides show improvement in morphology, and lack of phase separation. The result suggests that the introduction of organic peroxides such as DCP or BIB into PCL/PLA blend leads to cross-linking, and results in improving miscibility between phases. A similar effect can be observed for PCL/PLA blend compatibilized by PCL-*g*-PLA copolymer prepared via BIB as a free radical initiator. 

### 3.4. Thermal Stability 

**Figure 4:**
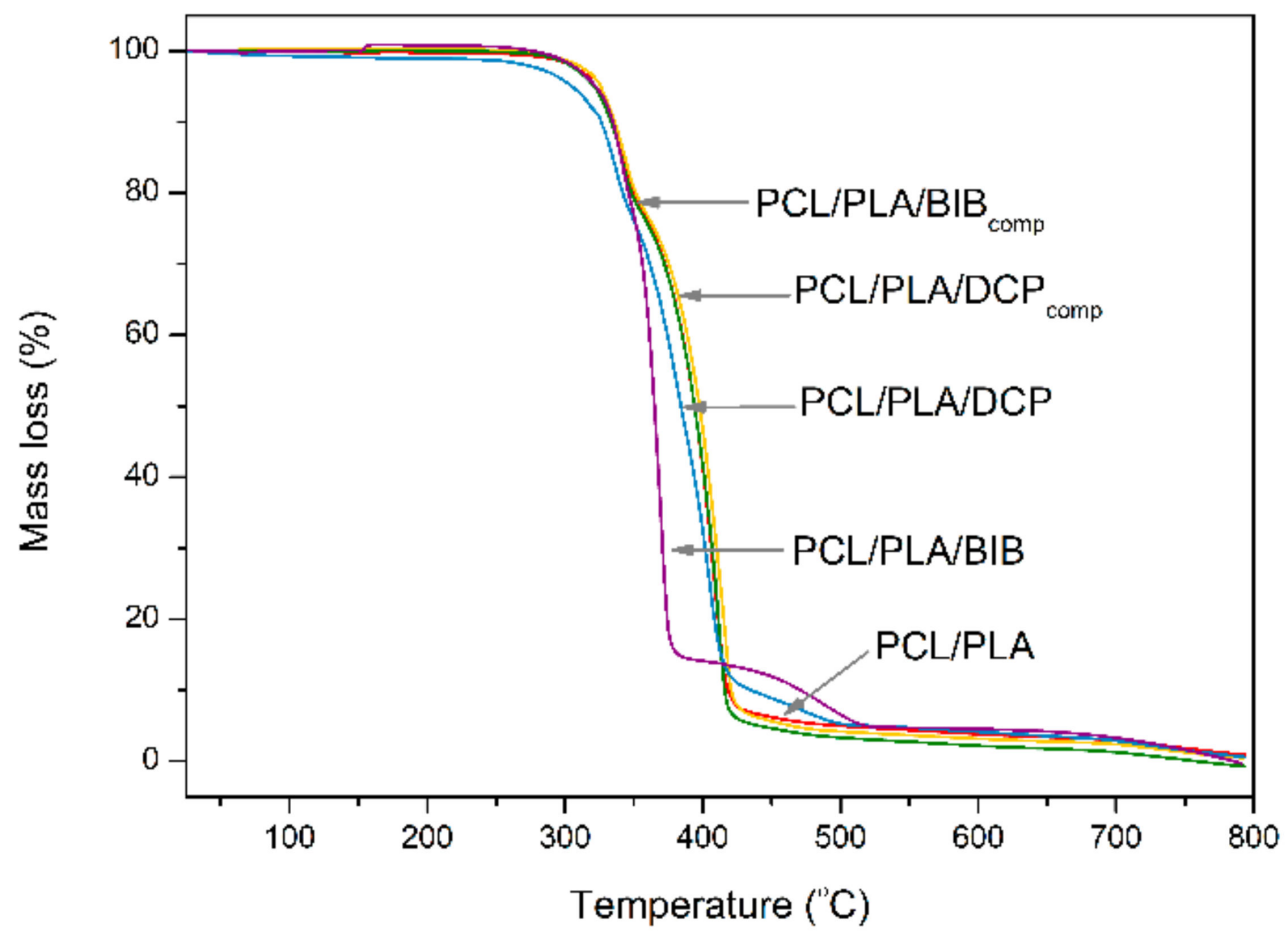
Results of TGA thermograms for PCL/PLA blends.

The thermal stability of the PCL/PLA blends investigated by thermogravimetric analysis (TGA) was presented in Figure 4 and listed in Table 1. The TGA curves show two mass loss steps corresponding well with the appropriate amounts of individual polymers mixed into the blends. The temperatures corresponding to the −2%, −5%, −10%, and −50% mass loss for PCL/PLA blends are essential for evaluating their thermal stability and are summarized in Table 1. The introduction of DCP and BIB has no satisfactory effect on the thermal stability of the PCL/PLA blends; the *T*_−50%_ clearly decreased to 384 °C and 365 °C, respectively, while *T*_−50%_ was 394 °C for the reference PCL/PCL blend, whereas the *T*_−50%_ has not changed for PCL/PLA blends compatibilized by PCL-*g*-PLA copolymers. The thermal stability of PCL/PLA blends modified by organic peroxides was decreased, which can be related with excessive amount of BIB added into the blends. 

### 3.5. Thermal and Crystallization Properties

**Figure 5:**
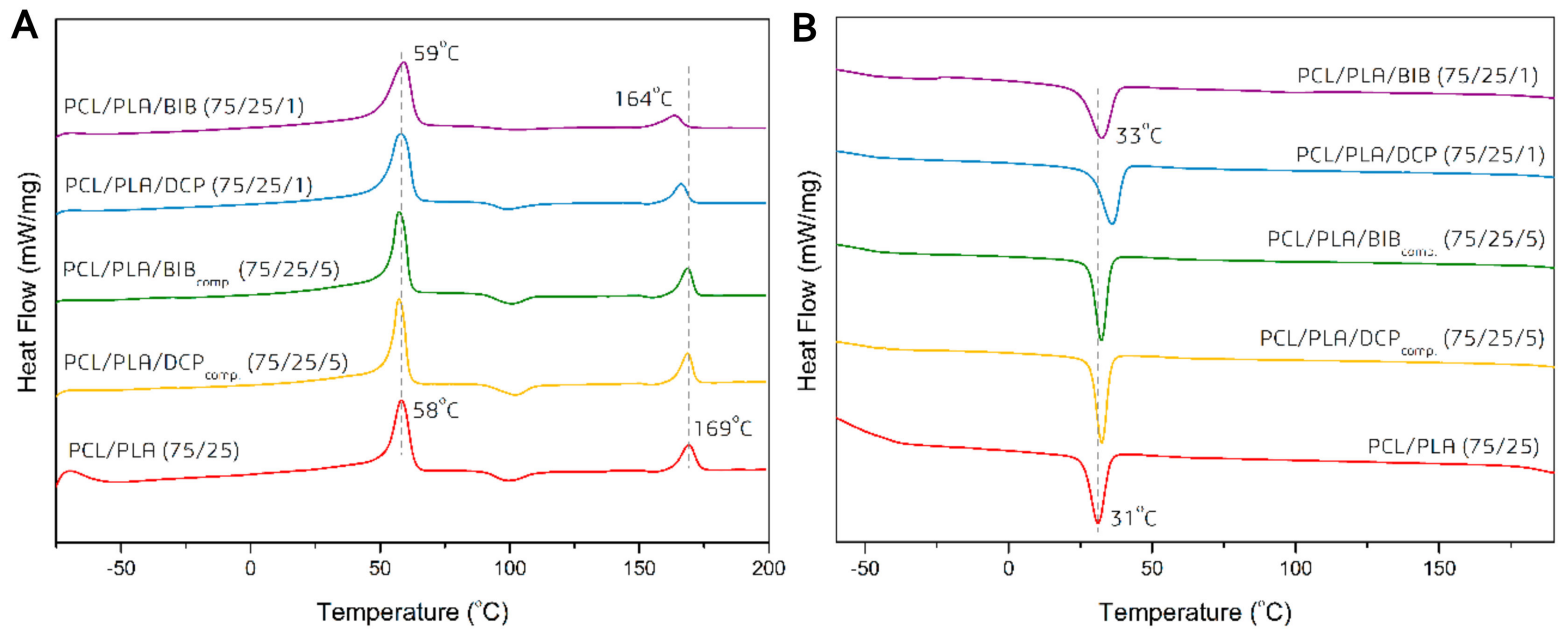
DSC second heating curves (**A**) and DSC cooling curves (**B**) of PCL/PLA blends.

Figure 5A,B shows the DSC second heating curves and cooling curves, respectively, of the PCL/PLA blends compatibilized by PCL-*g*-PLA copolymers or modified by organic peroxides. The detailed data of thermal parameters are listed in Table 2 including the melting temperature (*T*_m*(*PCL)_), melting enthalpy (Δ*H*_m(PCL)_), crystallization temperature (*T*_c*(*PCL)_), crystallization enthalpy (Δ*H*_c(PCL)_) and the degree of crystallinity (*X*_c(PCL)_) of PCL as well as melting temperature (*T*_m*(*PLA)_), melting enthalpy (Δ*H*_m(PLA)_), cold crystallization temperature (*T*_cc*(*PLA)_) and the degree of crystallinity (*X*_c(PLA)_) of PLA. The *T*_m_*, T*_c_ and *X*_c_ of unmodified PCL-rich blends were 58.2 °C, 31.2 °C, and 30.1% respectively. These mentioned parameters have not changed significantly for PCL/PLA blends compatibilized by PCL-*g*-PLA copolymers. The Δ*H*_m(PCL)_ value for PCL/PLA blends modified by organic peroxides increased to 37 J/g from 31 J/g for unmodified PCL/PLA blend. These results suggest that organic peroxides can promote the crystallization process of PCL, as also confirmed by *X*_c(PCL)_, which increased to 36% for modified PCL/PLA blends. However, the application of the organic peroxides causes decreasing of Δ*H*_m(PLA)_ value, which may indicate that peroxides can more intensively cross-link of PLA phase. It was also confirmed by *X*_c(PLA)_, which decreased to 34% and 30% for PCL/PLA blend modified by DCP and BIB, respectively. 

Furthermore, the melting point of PCL is overlapped with a glass transition of PLA, and it is not visible at the thermograph. The *T*_m_ of PLA shifted towards a lower temperature and *T*_m_ of PCL to a higher temperature for the PCL/PLA blends modified by DCP or BIB were observed. This effect confirms improvement in the interaction between PCL and PLA phases; which is suggested also by SEM images (Figure 3).

### 3.6. Dynamic Mechanical Analysis (DMA)

**Figure 6:**
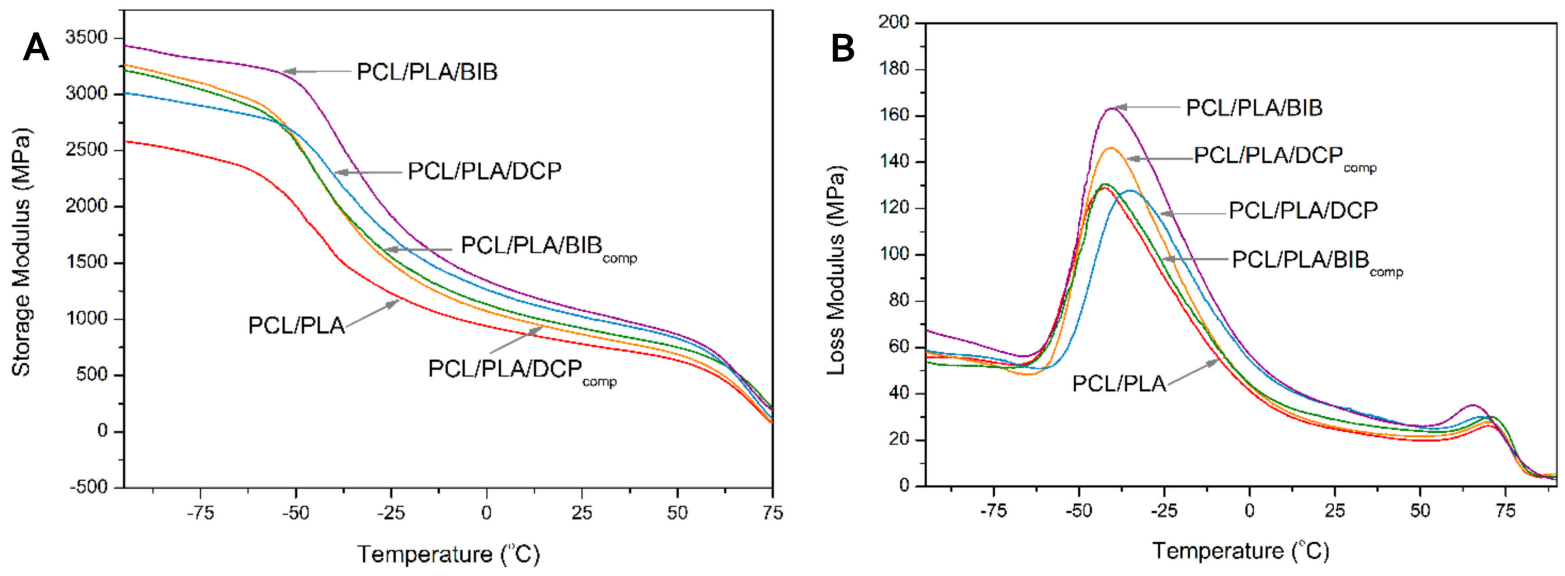
Storage (**A**) and Loss (**B**) Modulus of PCL/PLA blends.

Table 3 summarizes the glass transition temperature (*T*_g_), which corresponds to the maximum peak temperature of the loss modulus for neat PCL, neat PLA, and PCL/PLA blends. Figure 6 shows the storage and loss modulus curves of PCL/PLA blends. The unmodified PCL/PLA exhibited two peaks at −42 °C and 70 °C, attributed to the glass transition of PCL and PLA, respectively. The *T*_g_ (−42 °C) of PCL shifted to *T*_g_ (−35 °C) and *T*_g_ (−40 °C) for PCL/PLA blends with DCP and BIB added, respectively. The *T*_g_ (70 °C) of PLA in unmodified PCL/PLA blend was slightly higher than the *T*_g_ (65 °C) of PLA in the PLA/PCL blends modified by BIB, indicating that there was some better molecular interaction between the two components due to the cross-linking or branching reaction. The *T*_g_ parameters for both PCL and PLA have not changed for the PCL/PLA blends compatibilized by copolymers. 

Furthermore, the storage modulus (*E*’) increased for all tested PCL/PLA systems compared to the neat PCL, for which it was equal to 546 MPa. In the case of the PCL/PLA blends compatibilized by copolymers, the increase reached around 10% and 20% compared to unmodified PCL/PLA, and for the blends modified by reactive blending, it reached 30% and 40% depending on the peroxide used. Its increase may indicate compatibilization and interaction to PLA disperse phase.

### 3.7. Physico-Mechanical and Rheological Properties

**Figure 7:**
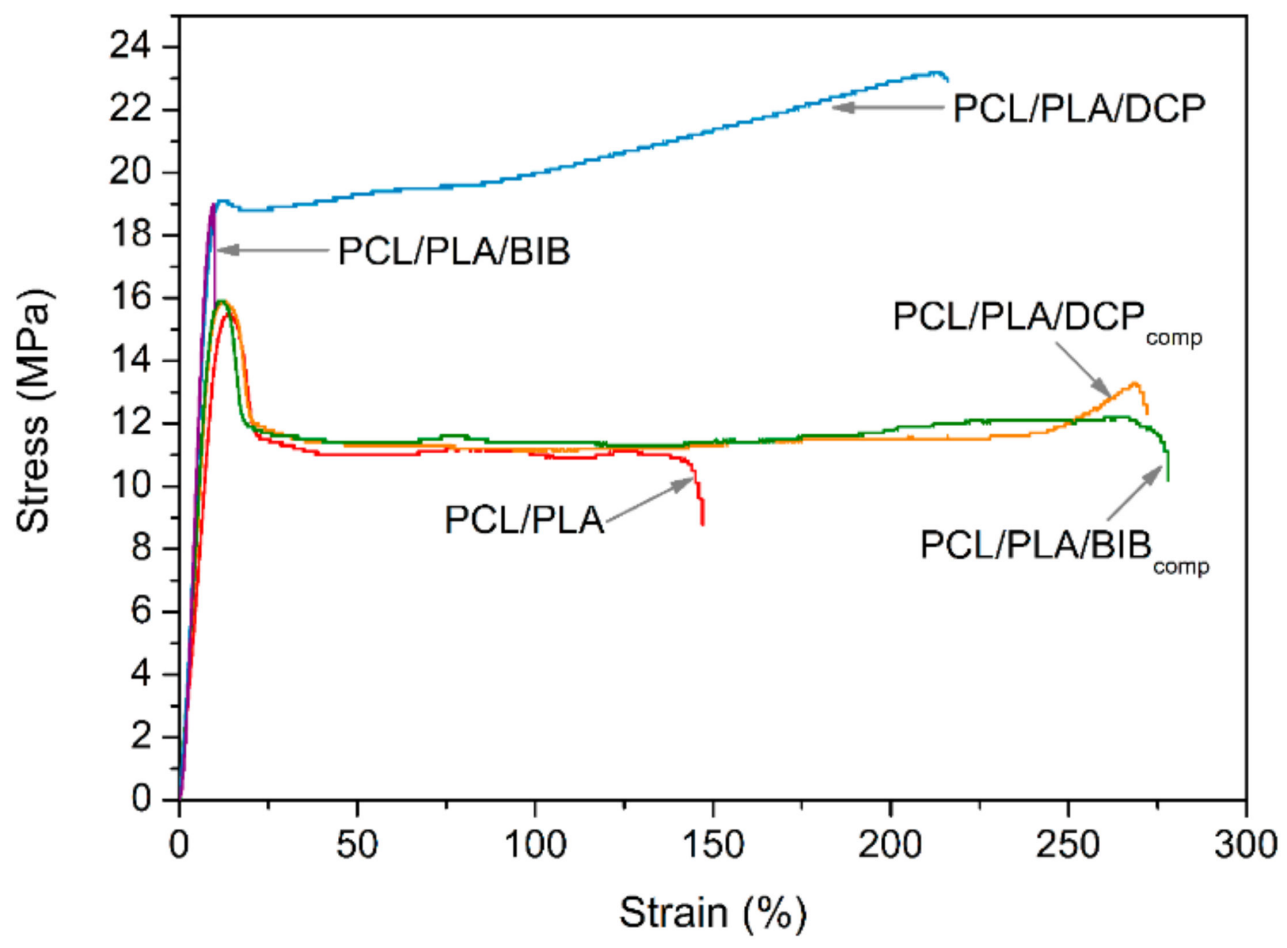
Representative stress-strain curves of studied PCL/PLA blends.

The stress-strain curves of the PCL/PLA blends are illustrated in Figure 7, and the corresponding results are listed in Table 4. The PCL/PLA blends compatibilized by PCL-*g*-PLA copolymers, which were prepared in solution, displayed a maximum fracture strain of about 220% or 240%, while for reference PCL/PLA blend the value was 160%. Comparing that with copolymers added in physical mixing, the fracture strain of blend modified by BIB in reactive processing was at least twenty times lower, and it was related to over cross-linking. The PCL/PLA blends modified by organic peroxides in reactive processing were characterized by higher yield stress and hardness. The organic peroxides processed in higher temperature can evolve a bigger number of free radicals and can lead to faster cross-linking of polymers not only branching or grafting.

Table 4 shows the brittleness parameter (*B*) of PCL/PLA blends calculated from the results of static and dynamic mechanical analysis according to Equation (2) proposed by Brostow et al. [22]. In the case of PLA homopolymer, which is characterized by high storage modulus and low elongation at break, the brittleness should be substantial. However, introduction of 25% PLA to PCL matrix does not significantly affect the brittleness of PCL/PLA blends, whereas the compatibility of these blends further reduces brittleness. Only the use of highly reactive BIB peroxide increases brittleness of PCL/PLA blend as a result of cross-linking. Materials that are brittle frequently have lower impact strength and higher stiffness properties.

Furthermore, the gel fraction correlated with melt flow index (MFI) for PCL/PLA blends presented in Table 4. The reference PCL/PLA blend, and compatibilized by PCL-*g*-PLA copolymers obtained in solution were completely soluble in chloroform, which proves that these blends did not show cross-linking. In case of PCL/PLA blend modified by BIB, the gel fraction was 72%, it confirms this blend is over cross-linking and causes a very limited ability to flow (not possible to measure by plastometer in studied conditions).

## 4. Conclusions

The PCL/PLA blends were reactively modified with 1.0 wt. % of DCP or BIB peroxides or compatibilized by PCL-*g*-PLA copolymers obtained in solution. The PCL-*g*-PLA copolymers act as compatibilizers, which are located at the interface of the immiscible PCL/PLA blends, improving the adhesion between two phases and the compatibility between the PCL and PLA. For PCL/PLA blends modified by organic peroxides, besides cross-linking, the PCL-PLA copolymers also can be created while preventing coalescence of PLA dispersed phase. However, the high temperature of the reaction environment and short time of decomposition of peroxides during reactive blending of PCL/PLA blends modified with DCP or BIB release free radicals immediately, which lead to more cross-linking of polymers than to copolymerization.

## Figures and Tables

**Table 1:** Thermal degradation parameters of PCL/PLA blends.

Samples	Weight Ratios	*T*_−__2%_ (°C)	*T*_−__5%_ (°C)	*T*_−__10%_ (°C)	*T*_−__50%_ (°C)	*R*_700 °C_ (%)
PCL/PLA	75/25	304	322	335	394	2.9
PCL/PLA/DCP_comp_	75/25/5	309	326	336	398	2.4
PCL/PLA/BIB_comp_	75/25/5	303	321	333	394	1.3
PCL/PLA/DCP	75/25/1	272	305	326	384	2.9
PCL/PLA/BIB	75/25/1	305	322	335	365	3.3

*T***_−_**_2%_, *T***_−_**_5%_*, T***_−_**_10%_*, T***_−_**_50%_ are the temperatures corresponding to the −2%, −5%, −10%, and −50% mass loss, respectively; and *R*_700_ is the residual weight at 700 °C.

**Table 2:** Thermal properties of PCL/PLA blends.

Samples	Weight Ratios	*T*_m*(*PCL)_ (°C)	Δ*H*_m(PCL)_ (J/g)	*T*_c*(*PCL)_ (°C)	Δ*H*_c(PCL)_ (J/g)	*T*_m*(*PLA)_ (°C)	Δ*H*_m(PLA)_ (J/g)	*T*_cc*(*PLA)_ (°C)	*X*_c(PCL)_ (%)	*X*_c(PLA)_ (%)
PCL/PLA	75/25	58.2	30.7	31.2	35.8	169.1	10.3	99.6	30.1	44.3
PCL/PLA/DCP_comp_	75/25/5	57.2	28.6	32.5	39.9	168.7	11.7	102.1	28.0	50.3
PCL/PLA/BIB_comp_	75/25/5	57.3	30.7	32.3	39.9	168.6	9.4	101.0	30.1	40.4
PCL/PLA/DCP	75/25/1	58.0	36.8	36.1	40.6	166.1	8.0	99.2	36.1	34.4
PCL/PLA/BIB	75/25/1	59.1	36.6	32.5	43.6	163.6	7.0	-	35.9	30.0

**Table 3:** DMA parameters of neat PCL, PLA, and PCL/PLA blends.

Samples	Weight Ratios	*E’_(_*_25 °C)_ (MPa)	*E”_(_*_PCL)_ (°C)	*E”_(_*_PLA)_ (°C)
PCL	100	546	−44.0	-
PCL/PLA	75/25	781	−42.0	70.0
PCL/PLA/DCP_comp_	75/25/5	867	−41.0	70.0
PCL/PLA/BIB_comp_	75/25/5	923	−42.0	70.0
PCL/PLA/DCP	75/25/1	1024	−35.0	68.0
PCL/PLA/BIB	75/25/1	1100	−40.0	65.0
PLA	100	3562	-	78.0

**Table 4:** Physico-mechanical and rheological properties of PCL/PLA blends.

Samples	Weight Ratios	Yield Stress (MPa)	Elongation at Break (%)	Hardness (ShD)	Gel Fraction (%)	MFI_170 °C/2.16 kg_ (g/10 min)	B 10^10^ %·Pa
PCL/PLA	75/25	15.4 ± 0.4	160 ± 65	56.8 ± 0.4	*soluble*	3.6 ± 0.3	0.080
PCL/PLA/DCP_comp_	75/25/5	15.7 ± 0.4	221 ± 59	58.0 ± 0.4	*soluble*	4.1 ± 0.3	0.052
PCL/PLA/BIB_comp_	75/25/5	16.1 ± 0.4	238 ± 35	58.3 ± 0.6	*soluble*	3.6 ± 0.3	0.046
PCL/PLA/DCP	75/25/1	19.4 ± 0.6	288 ± 68	59.7 ± 0.4	not measurable **	0.9 ± 0.2 *	0.034
PCL/PLA/BIB	75/25/1	19.9 ± 1.1	10.4 ± 0.4	59.9 ± 0.5	72.0 ± 4.0	-	0.87

* MFI measurement at 170 °C with load of 5 kg; ** dissoluble but formed a slurry that cannot be precisely weighted.
